# Visual biofeedback training reduces quantitative drugs index scores associated with fall risk

**DOI:** 10.1186/s13104-018-3859-7

**Published:** 2018-10-22

**Authors:** Eric Anson, Elizabeth Thompson, Samuel C. Karpen, Brian L. Odle, Edith Seier, John Jeka, Peter C. Panus

**Affiliations:** 1 Department of Otolaryngology, University of Rochester, 601 Elmwood Avenue, Box 629, Rochester, NY 14642 USA; 2 Department of Physical Therapy, Temple University, 1800 N Broad St, Philadelphia, PA 91222 USA; 3 University of Georgia College of Veterinary Medicine, 501 D. W. Brooks Drive, Athens, GA 30602 USA; 4 Pharmacy Practice, Gatton College of Pharmacy, East Tennessee State University, Box 70594, Johnson City, TN 37614 USA; 5 Mathematics and Statistics Department, College of Arts and Sciences, East Tennessee State University, Box 70663, Johnson City, TN 37614 USA; 6 Department of Kinesiology and Applied Physiology, University of Delaware, STAR Health Sciences Campus, 540 S College Ave, Newark, DE 19713 USA; 7 Pharmaceutical Sciences, Gatton College of Pharmacy, East Tennessee State University, Box 70657, Johnson City, TN 37614 USA

**Keywords:** Falls, Elderly, Polypharmacy, Biofeedback, Treadmill, Visual, Drug index, Community, Ambulatory, Covariate

## Abstract

**Objective:**

Drugs increase fall risk and decrease performance on balance and mobility tests. Conversely, whether biofeedback training to reduce fall risk also decreases scores on a published drug-based fall risk index has not been documented. Forty-eight community-dwelling older adults underwent either treadmill gait training plus visual feedback (+VFB), or walked on a treadmill without feedback. The Quantitative Drug Index (QDI) was derived from each participant’s drug list and is based upon all cause drug-associated fall risk. Analysis of covariance assessed changes in the QDI during the study, and data is presented as mean ± standard error of the mean.

**Results:**

The QDI scores decreased significantly (p = 0.031) for participants receiving treadmill gait training +VFB (− 0.259 ± 0.207), compared to participants who walked on the treadmill without VFB (0.463 ± 0.246). Changes in participants QDI scores were dependent in part upon their age, which was a significant covariate (p = 0.007). These preliminary results demonstrate that rehabilitation to reduce fall risk may also decrease use of drugs associated with falls. Determination of which drugs or drug classes that contribute to the reduction in QDI scores for participants receiving treadmill gait training +VFB, compared to treadmill walking only, will require a larger participant investigation.

*Trial Registration* ISRNCT01690611, ClinicalTrials.gov #366151-1, initial 9/24/2012, completed 4/21/2016

**Electronic supplementary material:**

The online version of this article (10.1186/s13104-018-3859-7) contains supplementary material, which is available to authorized users.

## Introduction

Polypharmacy is the clinical use of multiple drugs, and is a predictor of falls in the elderly [1–3]. Performance on mobility and balance tests decreases as the total number of drugs, or the number of drugs associated with fall risk, increases [3–6]. Additionally, reduction or elimination of fall-risk drugs from patients’ pharmacotherapy regimen improves physical function and reduces falls [7, 8]. Various clinical tools attempt to quantify the relationship between fall risk and drugs. Of these, Beer’s list and the Drug Burden Index are two of the most reported, yet both have limitations [9, 10]. The former is a prescriptive guideline identifying drugs associated with adverse effects in the elderly, but it does not quantitate drug-mediated fall risk. The latter quantitates drug-mediated fall risk, but is limited to drugs with sedation and anticholinergic adverse effects.

We developed a Quantitative Drug Index (QDI) that expands the list of drug adverse effects associated with fall risk [4]. The QDI is a clinically anchored index including all potential adverse effects associated with drug-mediated fall risk. To date there are no studies examining whether rehabilitation training to reduce falls influences the reported use of drugs associated with fall risk. We hypothesize that self-reported use of drugs associated with fall risk would decrease in participants receiving balance and mobility training on a treadmill while controlling trunk motion using visual feedback (+VFB), compared to those walking on a treadmill alone. The present research is an extension of our previously published efforts examining the effects of the QDI on balance and mobility testing [4].

## Main text

### Methods

This is a secondary analysis from a larger study examining the effects on balance and mobility of treadmill walking only, compared to treadmill gait training +VFB for controlling trunk motion [11]. The original study and this secondary analysis were approved by the institutional review boards at the University of Maryland, Temple University and East Tennessee State University. The purpose of the original investigation was to determine whether 4 weeks of treadmill gait training +VFB for controlling trunk motion would result in improved balance for older adults with self-reported balance problems. Participants were recruited for the study (ClinicalTrials.gov #366151-1) by advertisements in the newspaper and flyers placed at local retirement communities from 2012 to 2015. A total of 61 participants were evaluated for enrollment into the study. Eligibility criteria for the study included passing the mini-mental state examination (MMSE) with a score of 24 or higher and the ability to walk independently on a treadmill at a self-selected speed for 2 min [12]. The drug list information was provided by participants prior to and following the study period. All drugs were assessed and a QDI score for each participant’s drug list pre- and post-study calculated [4]. Differences between post- and pre-QDI scores were calculated for participants. Two participants were excluded from the study as they were unable to walk independently on the treadmill at a self-selected pace and one participant had an MMSE score of less than 24. A total of 58 consented participants were enrolled in the study. Six subjects did not complete the study, and the drug lists for four participants were not recorded, one at the beginning and three at the end of the study. Thus, for these secondary analyses, data from 48 participants were used. Twenty participants walked on a treadmill without VFB. Twenty-eight participants received treadmill gait training +VFB, with instructions to minimize trunk motion [11]. During enrollment age, height, weight and number of reported falls in the previous 12 months were recorded. Body Mass Index (BMI) was calculated from height and weight information. BMI could not be calculated for one participant in the treadmill only group as weight was not recorded for that individual. All participants also completed the activity-specific balance confidence (ABC) scale prior to and at completion of the study [13]. Graphics, t-test, analysis of covariance (ANCOVA), two way repeated measures analysis of variance (ANOVA) and descriptive analyses were conducted using IBM SPSS Statistics 22 (IBM Inc., Armonk, NY), or Slide Write (Advanced Graphics Software, Encinitas, CA) [14]. The randomization test comparing the QDI score differences between treadmill walking only and treadmill gait training +VFB was written in R: A Language and Environment for Statistical Computing (R Foundation for Statistical Computing, Vienna, Austria) [15]. The randomization test does not assume normality of the dependent variable, and was used to determine whether the t-test was biased by non-normality. Significance was set at = 0.05 for all analyses, and data presented as mean and standard error of the mean. The drug lists with corresponding QDI scores, age, MMSE scores, ABC scale scores, weight, height, reported falls in the previous 12 months and intervention group for all participants are in Additional file 1. The adverse effects for the QDI scale are in the supplemental data (Additional file 2: Appendix S1).

### Results

Prior to the study there were no significant differences between the treadmill walking only participants and treadmill gait training +VFB participants for age, BMI or number of reported falls within the previous 12 months (Table 1). The MMSE scores for two groups were also similar. The participants in the two groups at the beginning and end of the study recorded similar levels of balance confidence in performing various daily activities as determined by the ABC scale scores (Table 1).

**Table 1:** Comparison of participant parameters, reported falls, mental status and fall risk scores for both treadmill only participants and gait training +VFB participants

Variable	Pre-treatment scores		Post-treatment scores		p value
	Treadmill only	Gait training +VFB	Treadmill only	Gait training +VFB	
Age	75.6 ± 1.56	78.4 ± 1.19			0.15
BMI	27.2 ± 1.14	27.1 ± 1.34			0.94
MMSE	28.4 ± 0.28	28.4 ± 0.29			0.92
Falls	1.55 ± 0.71	1.14 ± 0.25			0.59
ABC scale	78.4 ± 3.58	75.0 ± 3.03	79.4 ± 4.09	75.0 ± 3.46	0.75

The sample size was N = 20 in the treadmill only group and N = 28 in the treadmill plus visual feedback (+VFB) group. One participant was lost for body mass index (BMI) calculation in the treadmill only group (N = 19), as weight was not recorded for the participant. Falls were the number of reported falls by participants within the previous 12 months. Two group T-test were conducted on the variables: age, body mass index (BMI), mini-mental status exam (MMSE) and reported falls in the previous 12 months. A 2-WAY repeated measures analysis of variance was conducted on the variable activity specific balance confidence (ABC) scale

**Fig. 1:**
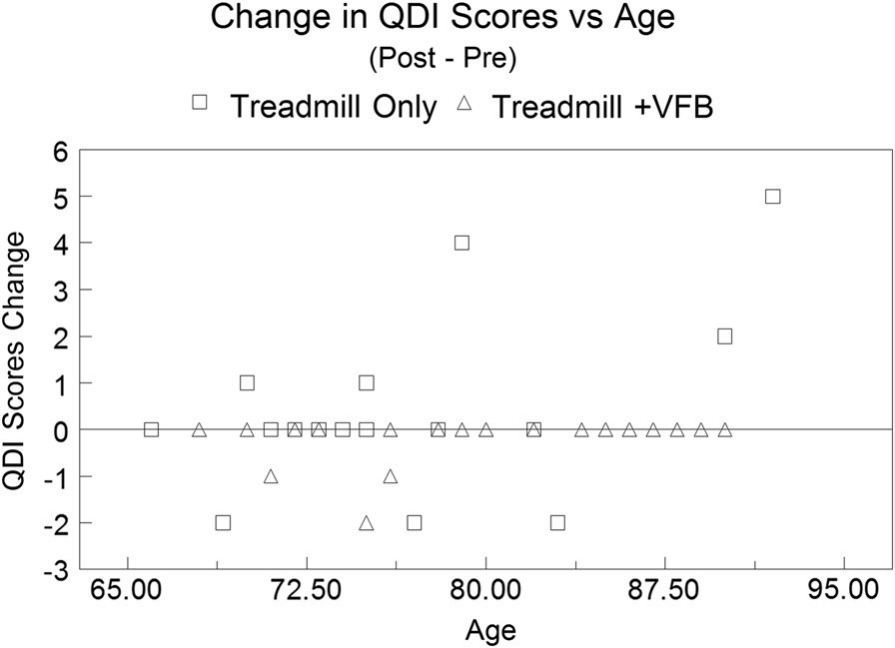
Scatterplot of the change in QDI scores for both the treadmill walking only participants (open squares) and treadmill gait training +VFB participants (open triangles), as a function of age. The horizontal line (score of zero), represents no change between the post- and pre- intervention QDI scores. The graph contains replicate change in QDI scores for several specific ages, in both treadmill walking only and treadmill gait training +VFB groups. These replicates may result in each icon representing more than a single response

A t-test of the intervention period changes in QDI scores between treadmill walking only (0.350 ± 0.386) compared to treadmill gait training +VFB (− 0.179 ± 0.090) was not significant (p = 0.13). The changes in QDI scores may have a non-normal distribution (Additional file 2: Appendix S2). To validate the t-test, a randomization test was done as it is immune to non-normality (Additional file 2: Appendix S3). The randomization test was also not significant (p = 0.14). As the two tests produced similar nonsignificant p-values, the t-test was assumed to be robust to non-normality. When age is included as a covariate (p = 0.007) in an ANCOVA, there was a significant difference (p = 0.031) for the changes in QDI scores during the intervention period when comparing treadmill walking only participants to treadmill gait training +VFB participants (Table 2). The average changes for the QDI score increased in the treadmill walking only participants, and decreased in the treadmill gait training +VFB participants. Finally, the treadmill walking only participants demonstrated no change, increased or decreased-QDI scores during the training period, whereas the treadmill gait training +VFB participants demonstrated either no change or decreased QDI scores during this period (Fig. 1). The post-treatment minus pre-treatment QDI scores for the majority of participants in both groups did not change during the training interval, as demonstrated by the change in QDI scores on the zero axis.

**Table 2:** Analysis of covariance for the change in QDI scores for treadmill walking only participants compared to treadmill gait training +VFB participants

Group	N	QDI scores change	ANCOVA	Age covariate (p value)
Treadmill walking only	20	0.463 ± 0.246	0.031	0.007
Treadmill gait training +VFB	28	− 0.259 ± 0.207		
Common average for age covariate				77.2

*QDI* Quantitative Drug Index, *VFB* visual biofeedback

Overall model was significant at p = 0.008. Change in QDI scores are post-scores minus pre-scores. Data is presented as mean and standard error of the mean. The common regression function variables were intercept equals − 5.595 and the slope for the age was 0.069. The coefficient of determination for the model was R^2^ = 0.194

### Discussion

A previous report indicated that withdrawal of participants’ fall-risk increasing drugs decreased subsequent fall risk, but not in the participants without pharmacotherapy intervention [7]. Surprisingly, a reduction of high fall risk cardiovascular drugs but not high fall risk psychotropic drugs significantly reduced subsequent falls. Unfortunately, the investigation did not use a scaled index for their drug-associated fall risk assessment, so quantitation was impossible. As the QDI assesses and quantifies fall risk associated with all adverse drug effects, the QDI should be able to detect drug-associated fall risk regardless of the pharmacologic mechanism [4]. In contrast, quantitative drug indices that do not assess all fall risk drug-associated adverse effects may miss fall risk associated with some drugs [5, 6, 9].

The current results demonstrate that participants in the treadmill gait training +VFB reduced reported use of fall-risk associated drugs compared to participants only walking on a treadmill. These results are based on a comparison of changes in QDI scores for both groups, after adjusting for age. Visualization of the QDI change score data demonstrates that several participants in the treadmill gait training +VFB group decreased use of drugs associated with fall risk, and no participant in this group added a drug associated with fall risk. Additionally, participant parameters for the two groups such as age and BMI were similar, as was mental status as determined by the MMSE scores. The number of reported falls in the previous 12 months were also similar for the two groups. The similarity in the number of reported falls by participants in both groups is also supported by similar self-reported balance confidence scores on typical daily life mobility tasks both before and after the training intervention. In aggregate, these results suggest that the two participant samples were similar based on measured physical, mental and mobility assessments. Thus, at present the most probable cause for the decrease in QDI scores, and use of fall-risk associated drugs, would be the treadmill gait training with visual biofeedback intervention. No previous investigation has documented that fall-risk reduction training affects self-reported use of drugs associated with fall risk.

Interestingly, the QDI scores for a number of participants in both groups remained unchanged during the intervention period. Pharmacotherapeutic management of the participants was not controlled during the study limiting causal understanding of this phenomenon. However, at least two competing hypotheses support the current results. First, participants without any change in QDI score may be more medically stable and thus less likely to have a medical encounter resulting in altered drug regimens. Thus the QDI would remain stable across the intervention. Second, although none of the collected participant characteristics differed between the groups analyzed, there may in fact be an unmeasured participant characteristic which would identify individuals more likely to report change in drug use during rehabilitation. The identification of participant characteristic(s) that predict changes in pharmacotherapy during rehabilitation, especially a decrease, would be of significant clinical value.

### Conclusions

This is the first brief report documenting that treadmill gait training with visual biofeedback may lead to decreased usage of fall-risk associated drugs. No other measured variables associated with fall risk were significantly different between the two groups of participants that could have accounted for the changes in the QDI scores during the study period. The mechanism(s) by which the treadmill gait training plus visual biofeedback mediates the decreased use of fall risk associated drugs will require further investigation in a larger population.

## Research limitations

There are several significant limitations to the current brief report. The sample size was small with only 48 participants. Additionally, only 12 out of 48 participants (25%) across both groups demonstrated a changes in their QDI scores. A longer training period may have increased this percentage. Only two time points were assessed. Multiple follow-up time points may strengthen the current results. However, pharmacotherapy for the participants is likely to change continuously making the validity of these additional time points questionable. The sample size is also insufficient to identify specific drugs or drug class(es) that resulted in the decrease in QDI scores for the treadmill gait training +VFB participants.

## Additional files

**Additional file 1.** Sheet 1: Mod Pre Med Data: includes a list of drugs for participants prior to the study with Quantitative Drug Index (QDI) scores under each drug. Total QDI score for each participant is in column AJ, and total drug counts for each participant is in column AK. Sheet 2: Mod Post Med Data: includes a list of drugs for participants at the end of the study with Quantitative Drug Index (QDI) scores under each drug. Total QDI score for each participant is in column AD, and total drug counts for each participant is in column AE. Sheet 3: Pre & Post data: columns for each variable are as described: total pre-study QDI drug score (Mod Pre Drug Score) for each participant, total post-study QDI drug score (Mod Post Drug Score) for each participant, change in QDI scores (dQDI) post-intervention minus pre-intervention, whether participants (Group) were in the control arm (treadmill walking only—0) or the intervention arm (gait training plus visual biofeedback—1); age, height, weight, participant stated number of falls in the previous 12 months (fall 12 prev months), Mini-Mental State Exam scores (MMSE), pre-study Activity Specific Balance Confidence scale scores (Pre ABCscore) and post-study Activity Specific Balance Confidence scale scores (Post ABCscore).

**Additional file 2: Appendix S1.** Table and Text of Quantitative Drug Index adverse effects. **Appendix S2.** Figure and text documenting non-normal distribution of QDI scores. **Appendix S3.** Figure of randomization analysis and supporting text.

## Abbreviations

ABC: activity-specific balance confidence
ANCOVA: analysis of covariance
ANOVA: analysis of variance
BMI: body mass index
MMSE: mini-mental state examination
QDI: Quantitative Drug Index
+VFB: plus visual feedback
