# Supplementary material for: Visual biofeedback training reduces quantitative drugs index scores associated with fall risk
Source: BMC Res Notes. 2018 Oct 22;11:750. doi: 10.1186/s13104-018-3859-7 (PMC6196457; doi:10.1186/s13104-018-3859-7)

Additional data

Appendix S1. List of all adverse effects for the QDI in this investigation that were scored as associated with falls. All adverse effects were weighted equally.

| accidental injury | dizziness | orthostatic hypotension |
| --- | --- | --- |
| arthralgia | drowsiness | sedation |
| ataxia | falling | skeletal weakness |
| blurred vision | fatigue | somnolence |
| catatonic states | hypoglycemia | syncope |
| cognitive dysfunction | hypotension | tremor |
| confusion | incoordination | unsteady gait |
| coordination impaired | lethargy | vertigo |
| delirium | lightheadedness | visual disturbances |
| diplopia | muscle weakness | weakness |
| disoriented | myalgia |  |

Appendix S2. Dot plot of the distribution of change in QDI scores (Post-Pre QDI Scores) for both the treadmill walking only participants (Control, N=20), and the treadmill gait training + VFB participants (Experimental, N=28).


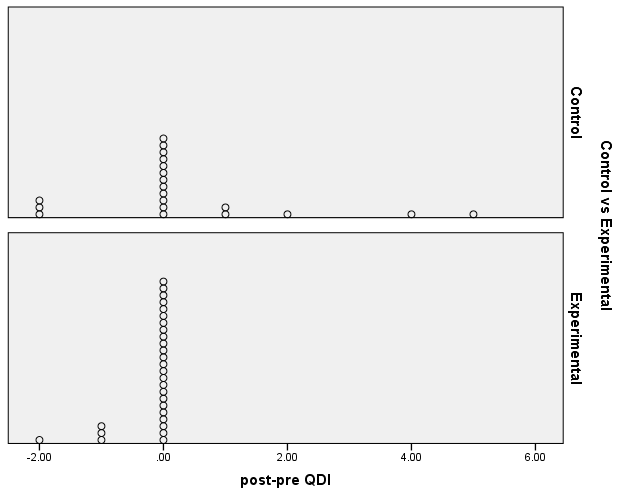


Appendix S3. Results of 10,000 randomizations examining the changes in QDI scores between the treadmill walking only participants and treadmill gait training +VFB participants. The randomization testing demonstrated that the two groups were not significantly (p= 0.14) different. The score (0.529), and associated red lines, represents the difference between the two groups and its value multiplied by -1 because the alternative hypothesis is two sided. The values depicted in the histogram are values taken by the difference for 10,000 random re-resampling of the data in two groups. In 14% of those regroupings the difference was either greater than 0.529 or lower than -0.529 indicating that the difference between treadmill walking only and treadmill gait training +VFB is not extreme enough to reject the null statistical hypothesis.


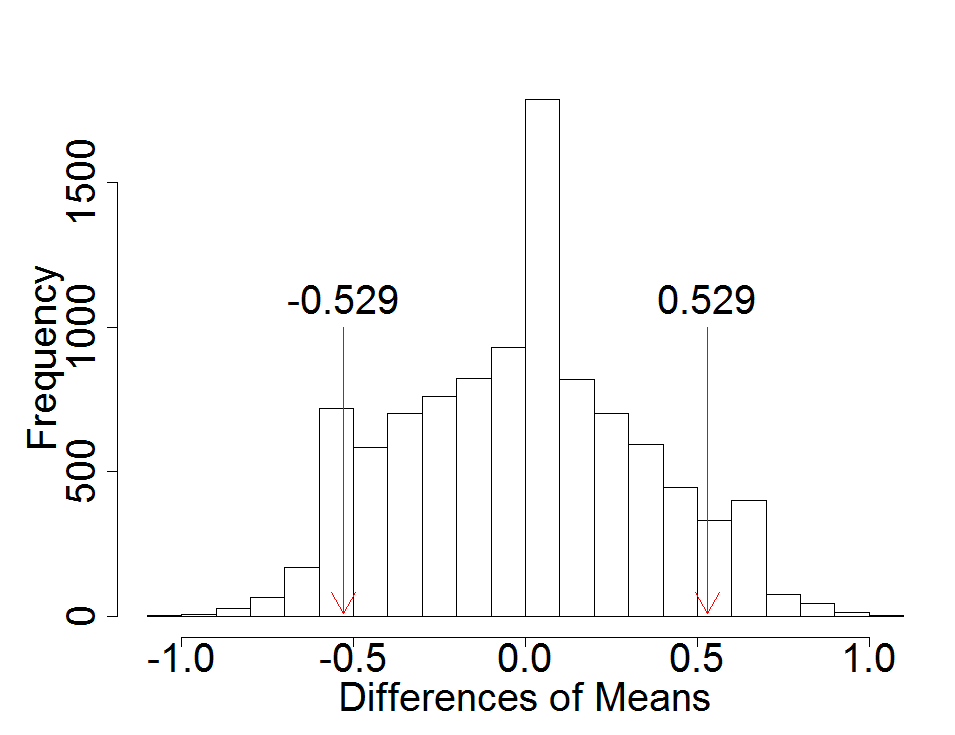

Supplement: Supplementary file 2 — Additional file 2: Appendix S1. Table and Text of Quantitative Drug Index adverse effects. Appendix S2. Figure and text documenting non-normal distribution of QDI scores. Appendix S3. Figure of randomization analysis and supporting text. [file 13104_2018_3859_MOESM2_ESM.docx]
